# Integration of Spectral Reflectance across the Plumage: Implications for Mating Patterns

**DOI:** 10.1371/journal.pone.0023201

**Published:** 2011-08-10

**Authors:** Miklós Laczi, János Török, Balázs Rosivall, Gergely Hegyi

**Affiliations:** Behavioural Ecology Group, Department of Systematic Zoology and Ecology, Eötvös Loránd University, Budapest, Hungary; Arizona State University, United States of America

## Abstract

**Background:**

In complex sexual signaling systems such as plumage color, developmental or genetic links may occur among seemingly distinct traits. However, the interrelations of such traits and the functional significance of their integration rarely have been examined.

**Methodology/Principal Findings:**

We investigated the parallel variation of two reflectance descriptors (brightness and UV chroma) across depigmented and melanized plumage areas of collared flycatchers (*Ficedula albicollis*), and the possible role of integrated color signals in mate acquisition. We found moderate integration in brightness and UV chroma across the plumage, with similar correlation structures in the two sexes despite the strong sexual dichromatism. Patterns of parallel color change across the plumage were largely unrelated to ornamental white patch sizes, but they all showed strong assortative mating between the sexes. Comparing different types of assortative mating patterns for individual spectral variables suggested a distinct role for plumage-level color axes in mate acquisition.

**Conclusions/Significance:**

Our results indicate that the plumage-level, parallel variation of coloration might play a role in mate acquisition. This study underlines the importance of considering potential developmental and functional integration among apparently different ornaments in studies of sexual selection.

## Introduction

Many animal species develop conspicuous traits, such as acoustic and visual cues, and display multiple secondary sexual ornaments [1]. Multiple traits can enhance the reliability of information, so animals may use more than one signal simultaneously to assess the quality of potential mates or rivals [2]–[4]. Numerous bird species possess differently colored plumage patches, for example black, white, green and yellow in great tits (*Parus major*), or blue and reddish brown in eastern bluebirds (*Sialia sialis*). The countless kinds of bird plumage colors arise from two primary mechanisms. Pigment-based colors result from light absorption by pigments (mainly melanins and carotenoids), while structural colors are produced by the feather nanostructure that scatters the incident light [5]. These two mechanisms usually work together [6], [7]. The exception is achromatic white color which is purely structural and – in contrast to chromatic (ultraviolet (UV), blue, green) structural colors – it results from incoherent scattering [5].

Several lines of evidence suggest that color traits can be under sexual selection and convey information about individual quality. In contrast to carotenoid-based ornaments that signal physical condition and depend on environmental factors [8], melanin-based traits may reflect viability, genetic quality [9], [10] (but see [11]) or social status [12], and are often independent of condition (e.g. [13]). However, the synthesis of melanin pigments has high energetic costs [14], and melanin-based ornaments may also be influenced by environmental and physiological factors (e.g. [15], [16]). Chromatic structural colors have been found to indicate viability [17], parental effort [18], parasite load [19], territory quality [20] and offspring sex ratio [21]. Their expression may also depend on nutritional condition [22], [13] and molt duration [23]. The reliability of information from depigmented white patches originates from their high maintenance costs: white areas are more fragile [24], less resistant against feather-degrading bacteria [25], preferred by feather lice [26], and they also enhance predation risk [27] and intrasexual aggression [28]. Moreover, achromatic structural color can be condition-dependent [29]. Although it is well known that size of depigmented patches can indicate individual quality and influence mate choice (e.g. [30], [31]), the spectral properties of white ornaments have been poorly studied, and there are much fewer results supporting their function as signals. For example, white intensity in male black-capped chickadees (*Poecile atricapillus*) is related to the proportion of within-pair nestlings [32], social dominance and female choice [33]. White intensity also indicates immune defense in female common eiders (*Somateria mollissima*) [34]. In northern pintails (*Anas acuta*), a study demonstrated female preference for males with a whiter breast [35]. Bridge & Eaton [36] found in three species of tern (*Sterna*) that UV chroma and brightness were lower in primaries that had molted fewer times and were therefore older, which may play a role in mating decisions. Finally, the achromatic intensity of the white wing bar in house sparrows (*Passer domesticus*) was affected by their molt speed [37].

Unfortunately, partly because of methodological considerations, most published studies of this sort paid attention to only one single aspect of ornamentation [38], [39], even where the study species expressed more than one attractive character, although there has been growing interest in investigating multicomponent signals [31], [40]–[45]. The currently dominant view is that color types of different origin convey different information (e.g. [13]) and are controlled by different developmental processes [8], [46], [47], which may have contributed to the consideration of these characters in separate analyses [32]. Even distinct plumage patches of the same color type are often treated as independent traits (e.g. [22]), regardless of their potential functional or developmental similarities.

In reality, it may often prove difficult even to define individual ornaments with respect to their signal content if multiple conspicuous traits in fact constitute a composite signaling system. For example, sexual selection may act towards maximizing the condition-dependence of distinct components of the phenotype and the different ornaments may thereby evolve to share developmental pathways and regulatory mechanisms [48]. Therefore, even if some traits correlate with distinct single aspects of individual quality, these traits may still interrelate due to dominant determinants of quality [3], [49]. Moreover, receivers capable of processing a composite system of several individual ornamental traits may benefit in many different ways, including the acquisition of more accessible information, more reliable information, or even emergent information not conveyed by any individual component trait [50]. Although there are some studies that have examined more than one color ornament simultaneously e.g. [14], [43], [45], most of these treated the given characters as separate units and not as a composite system (but see [18], [44], [51]).

When tracking sexual selection in a system of potentially correlated ornamental traits, the first step is to explore the interrelations of these traits, and the subsequent analyses of the information content and use of ornamentation should be performed according to these results. If there is little interdependence among the investigated characters, the different color traits can be analyzed in isolation. If there is significant interdependence, the possibility of a functionally integrated signal system should be considered. Here we used spectral data from collared flycatchers (*Ficedula albicollis*) to examine the integration of reflectance attributes among plumage areas and the potential role of whole plumage reflectance variation in mate acquisition. We first constructed correlation matrices of area-level reflectance descriptors (brightness and UV chroma) and evaluated these matrices to determine the strength and level of color integration among plumage areas. We also compared the structure of correlation matrices between the sexes using a matrix similarity hierarchy approach to see whether male and female data can be analyzed together. Based on these results, we fitted principal component analyses (PCAs) of reflectance descriptors across the plumage, and assessed the parallelism of integrated, plumage-level reflectance variation with the sizes of ornamental patches, representing another potential level of plumage signal integration. Finally, we looked for assortative mating (indicating mutual sexual selection [52]) in relation to plumage-level color axes, and used a meta-analytic procedure to estimate the relative roles of area-specific versus plumage-level color variation in putative sexual selection processes. Although there have been a few studies that treated color traits as a composite system [44], [51], no study to our knowledge has investigated mating patterns while considering the reflectance of different plumage parts as an integrated ornament system. Analyses of trait correlation structure and direct or indirect analyses of sexual selection for composite traits are complementary and are difficult to interpret separately. First, examining trait correlation structure but not sexual selection is problematic because receivers may or may not consider trait integration in their mating decisions, so we may be describing functionally neutral patterns. Second, examining mating patterns for trait complexes without quantifying trait correlation structure may lead to artifacts because the subjectively outlined “complexes” for which we find significant mating patterns may not in fact exist.

Collared flycatchers have composite plumage ornamentation with melanin-pigmented dark and depigmented white parts. Breeding males display non-iridescent black plumage with white underparts, collar, forehead patch and wing patches, while females show greyish-brown plumage with white underparts and wing patches, and lack the collar and usually also lack a measurable forehead patch ([53] and our personal observations). Based on human-visible differences, we predicted that the sexes would differ not only in the spectral traits of pigmented parts (browner in females), but also in those of the depigmented ones (duller in females). We also predicted coloration to also differ between ages in males (yearlings have visibly duller wing color [53]). Since all plumage areas in our study species are either melanin-pigmented or depigmented and therefore all share at least one color production mechanism (melanin and structural, or purely structural), we expected that interdependence of reflectance among different plumage parts will lead to a few main dimensions of coloration.

In the case of strong color integration across the plumage and a similar information content and role of plumage color in the two sexes, we expected positive assortative mating in relation to the main axes of overall plumage color (composite color axes). However, such assortative mating may indicate that 1) receivers use multiple correlated signals separately, or that 2) they consider these signals together as an integrated ornamentation system. Therefore, we designed additional analyses to establish the meaning of assortative mating for overall plumage color. In case the receivers functionally integrated the individual, area-level signals and used them as a single overall indicator of quality, we expected that individual spectral features (area-level brightness and UV chroma), the building blocks of our composite color axes, would show stronger assortative mating within the detected main axes of reflectance variation than among these, but the within-axis assortative mating would be trait-independent. In other words, if variables A and B were parts of one composite axis but C was not, we expected that the correlation between A in males and A in females would have the same magnitude as that between A in males and B in females, but both would be stronger than that between A in males and C in females. This trait-independence is critical: within a composite trait, if assortative mating within an area-level variable is systematically stronger than that between two variables, this would suggest that receivers pay attention to multiple independent traits rather than, or in addition to, integrating them.

## Results

### Sexual dichromatism

**Figure 1:**
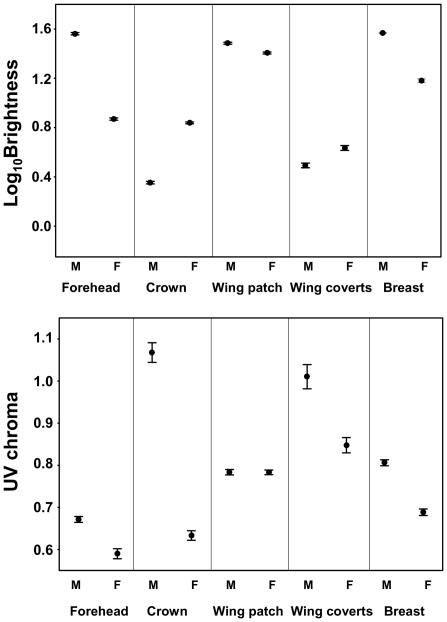
Sex comparisons of brightness and UV chroma for five plumage areas in collared flycatchers. Means±SE. Males (M), females (F). The analyses were performed on year-standardized data, but the figures are based on unstandardized data to better show the absolute extent of the differences.

We found strong sexual dichromatism (which is readily visible for humans) not only in the pigmented areas of the plumage but also in the depigmented areas. Except for the UV chroma of the white wing patch, area-specific spectral variables differed significantly between sexes (n = 125 females and 127 males; p = 0.78 in wing patch UV chroma, and p<0.0001 in the other nine cases; Fig. 1). Males had more pronounced UV chroma irrespective of the type of color considered (i.e. melanized or white area). As also visible to the human eye, females had brighter pigmented areas than males, but in contrast to this, their white areas had a lower brightness.

### Correlation structure of spectral variables

The correlation analyses revealed moderate integration among different parts of the plumage regarding both brightness and UV chroma (Table 1). 5 of 10 and 7 of 10 correlations between brightness variables from different plumage areas were significant for females and males respectively (unsigned mean r±SE = 0.21±0.01 in females and 0.14±0.01 in males), while for UV chroma the corresponding ratios were 6 of 10 and 10 of 10 (unsigned r = 0.22±0.02 in females and 0.29±0.01 in males). Correlations between brightness and UV chroma, on the other hand, were significant in only 3 of 25 cases in females (unsigned r = 0.09±0.001) and 4 of 25 cases in males (unsigned r = 0.09±0.003). When the sexes are analyzed together as prompted by their similar correlation structures (see next section), the ratios of significant correlations are 8 of 10 for brightness and 10 of 10 for UV chroma, but only 2 of 25 between brightness and UV chroma (unsigned r = 0.18±0.01, 0.25±0.01 and 0.06±0.002, respectively). Both of the non-significant correlations for brightness were caused by the plumage area (i.e. wing coverts), which itself formed a separate principal component (PC; see below). These results suggest that multiple brightness and multiple UV chroma traits are to some extent integrated across plumage areas, but the integration between brightness and UV chroma is very weak. To see whether the moderate correlation strengths we observed for brightness and UV chroma separately were suitable for dimension reduction by PCA, we calculated the Kaiser-Meyer-Olkin (KMO) index of factor sampling adequacy ([54], see Methods for details).This test examines whether the magnitude of information loss by using integrated measures permits the statistical integration of traits or not. Values of this index (sexes separately and together) were well above the acceptable level of 0.5 and indicated moderate integration (0.571 to 0.632 for brightness, 0.581 to 0.706 for UV chroma). This indicates that dimension reduction by PCA is a reasonable decision. In accordance with these results, we performed the PCA separately for brightness and for UV chroma.

**Table 1:** Pearson correlations of spectral variables in collared flycatcher females and males.

Sex	Spectral variable	Wing patch UV	Wing coverts B	Wing covert UV	Forehead B	Forehead UV	Crown B	Crown UV	Breast B	Breast UV
**Female**	**Wing patch B**	−0.10	−0.05	−0.18*	0.29***	0.07	0.26**	0.08	0.27**	0.01
**Female**	**Wing patch UV**		−0.11	0.19*	−0.01	0.15	−0.08	0.24**	0.04	0.12
**Female**	**Wing coverts B**			0.22*	0.05	−0.22*	0.20*	−0.16	−0.03	−0.21*
**Female**	**Wing covert UV**				−0.06	0.21*	−0.06	0.24**	−0.11	−0.07
**Female**	**Forehead B**					−0.12	0.57***	−0.05	0.21*	−0.02
**Female**	**Forehead UV**						−0.13	0.68***	0.02	0.12
**Female**	**Crown B**							0.03	0.28**	−0.04
**Female**	**Crown UV**								0.02	0.18*
**Female**	**Breast B**									0.09
**Male**	**Wing patch B**	−0.03	−0.14	0.22*	0.18*	−0.05	0.23**	−0.12	0.25**	0.04
**Male**	**Wing patch UV**		−0.17	0.26**	−0.10	0.30***	−0.09	0.23*	−0.04	0.34***
**Male**	**Wing coverts B**			−0.13	0.03	−0.02	0.11	**−0.22**	−0.10	−0.01
**Male**	**Wing covert UV**				−0.08	0.20*	0.14	0.44***	−0.05	0.30***
**Male**	**Forehead B**					−0.04	0.08	−0.28**	0.22*	0.05
**Male**	**Forehead UV**						0.08	0.24**	−0.15	0.38***
**Male**	**Crown B**							0.16	0.15	0.14
**Male**	**Crown UV**								−0.02	0.21*
**Male**	**Breast B**									−0.27**
**Pooled**	**Wing patch B**	−0.07	−0.10	0.03	0.25***	0.08	0.22***	0.00	0.27***	0.03
**Pooled**	**Wing patch UV**		−0.12	0.24***	−0.07	0.18**	−0.05	0.26***	−0.01	0.23***
**Pooled**	**Wing coverts B**			0.05	0.02	−0.06	0.15*	−0.08	−0.09	−0.09
**Pooled**	**Wing covert UV**				−0.06	0.24***	0.09	0.35***	−0.08	0.13*
**Pooled**	**Forehead B**					−0.01	0.24***	−0.13*	0.22***	0.04
**Pooled**	**Forehead UV**						0.01	0.42***	−0.04	0.27***
**Pooled**	**Crown B**							0.17**	0.21***	0.04
**Pooled**	**Crown UV**								0.01	0.17**
**Pooled**	**Breast B**									−0.07

Spectral data were standardized for year (to a mean of zero and a standard deviation of one). Significant correlations are marked with bold (* p<0.05; ** p<0.01; *** p<0.001).

### Similarity of spectral correlation structure between the sexes

We compared correlation matrices between the sexes using common principal component (CPC) analysis [55], which estimates the relative suitability of multiple different degrees of matrix similarity using a model hierarchy approach based on the Akaike Information Criterion (AIC; [56], see Methods for details). The similarity of the brightness correlation structure of plumage areas between males and females was best described by a partial CPC model with the first four components accepted as common. The second best model (AIC difference from best model, dAIC = 1.19) suggested only three common PC axes. Higher and lower levels of similarity were unsupported by our data (e.g. for equality, proportionality and unrelated structure of the correlation matrices, dAIC>10). In the case of UV chroma, the partial CPC model with the first three PCs shared between the female and male correlation matrices seemed most suitable given the data, while the model with the first two PCs accepted as common was the second best (dAIC = 2.55).

### Principal component analyses

**Figure 2:**
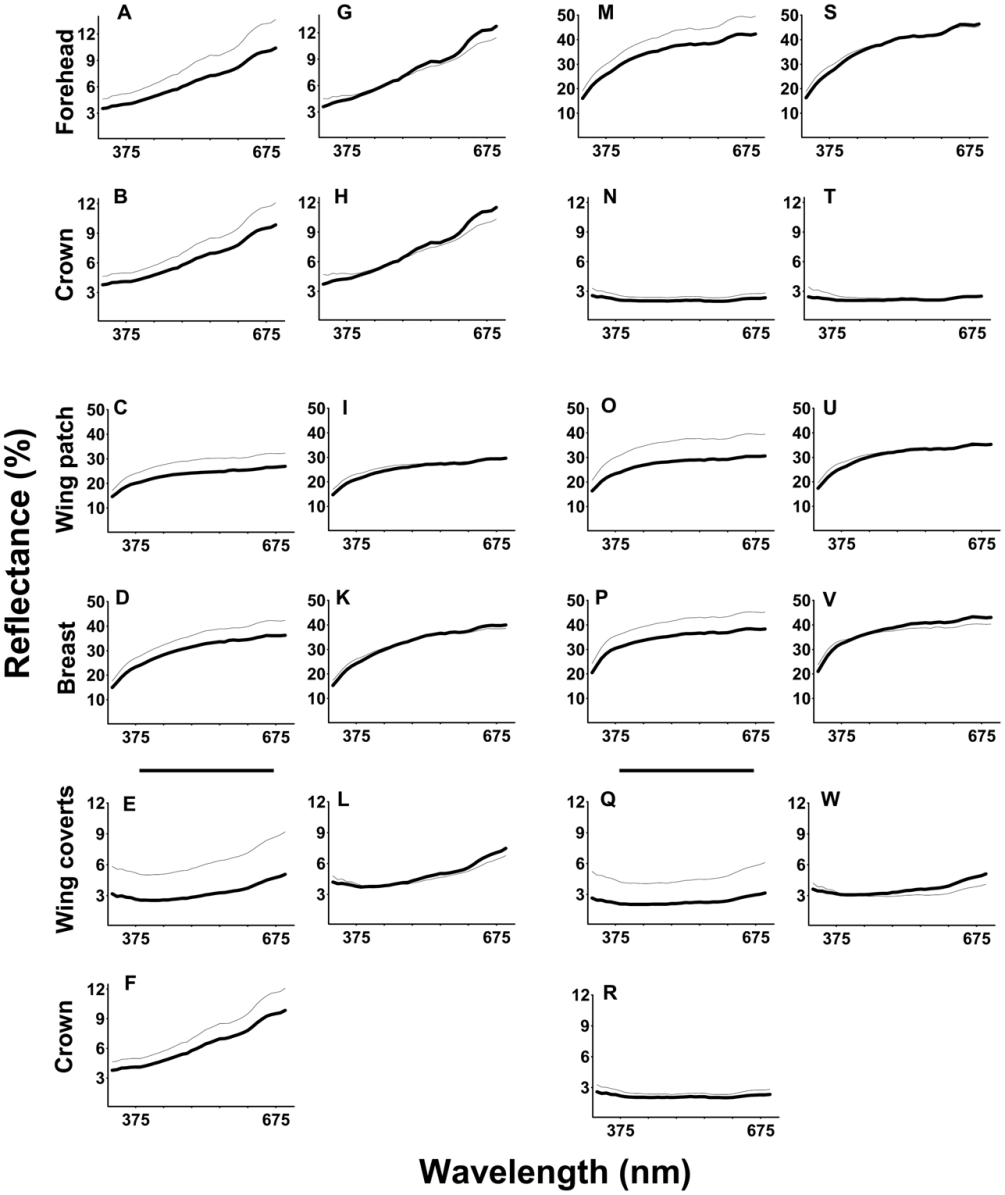
Mean reflectance values of individual wavelength bands for low versus high values of color PCs. This figure is an illustration of the meaning of our PCs without any assumption on color coding or visual system: the PCAs use brightness or UV chroma and not raw spectral information. Low (thick lines) and high values (thin lines) are coded relative to the overall mean. The two sexes are presented separately despite the pooled analysis because the raw spectral data we plot here show high sexual dichromatism. Female brightness PC1 (A–D), female brightness PC2 (E–F), female UV chroma PC1 (G–L), male brightness PC1 (M–P), male brightness PC2 (Q–R), male UV chroma PC1 (S–W).

Given that we found little correlation between brightness and UV chroma but similar covariance structures of brightness and UV chroma among plumage areas in females and males, we calculated separate PCAs for brightness and UV chroma but pooled data from the two sexes. Before this, the strong sexual dichromatism required the standardization of spectral variables within sexes (to a mean of zero and a standard deviation of one), or otherwise the PC axes would have largely explained spectral variation between the sexes. To illustrate (without any restrictive assumption) the meaning of the integrated plumage color axes we constructed in this step, Fig. 2 shows how the raw reflectance spectra of the five plumage parts in females and males changed along the brightness or UV chroma PC axes.

The first brightness PC (brightness PC1) loaded positively with wing patch, forehead, crown and breast brightness. Brightness PC2 loaded positively with the brightness of the wing coverts and that of the crown (Table 2). This means that an individual with higher brightness PC1 value has increased plumage brightness on its white areas and on its melanized head feathers (Fig. 2A–D, M–P), while a lower brightness PC2 value indicates darker melanized plumage parts (Fig. 2E–F, Q–R).

**Table 2:** Principal component loadings with the plumage color variables.

	Brightness PC1	Brightness PC2	UV chroma PC1
**Forehead**	0.66	0.10	0.69
**Crown**	0.62	0.44	0.74
**Wing patch**	0.68	−0.24	0.58
**Wing coverts**	−0.02	0.90	0.62
**Breast**	0.65	−0.24	0.51
**Expected variance (%)**	*34.0*	*22.5*	*40.3*

**Figure 3:**
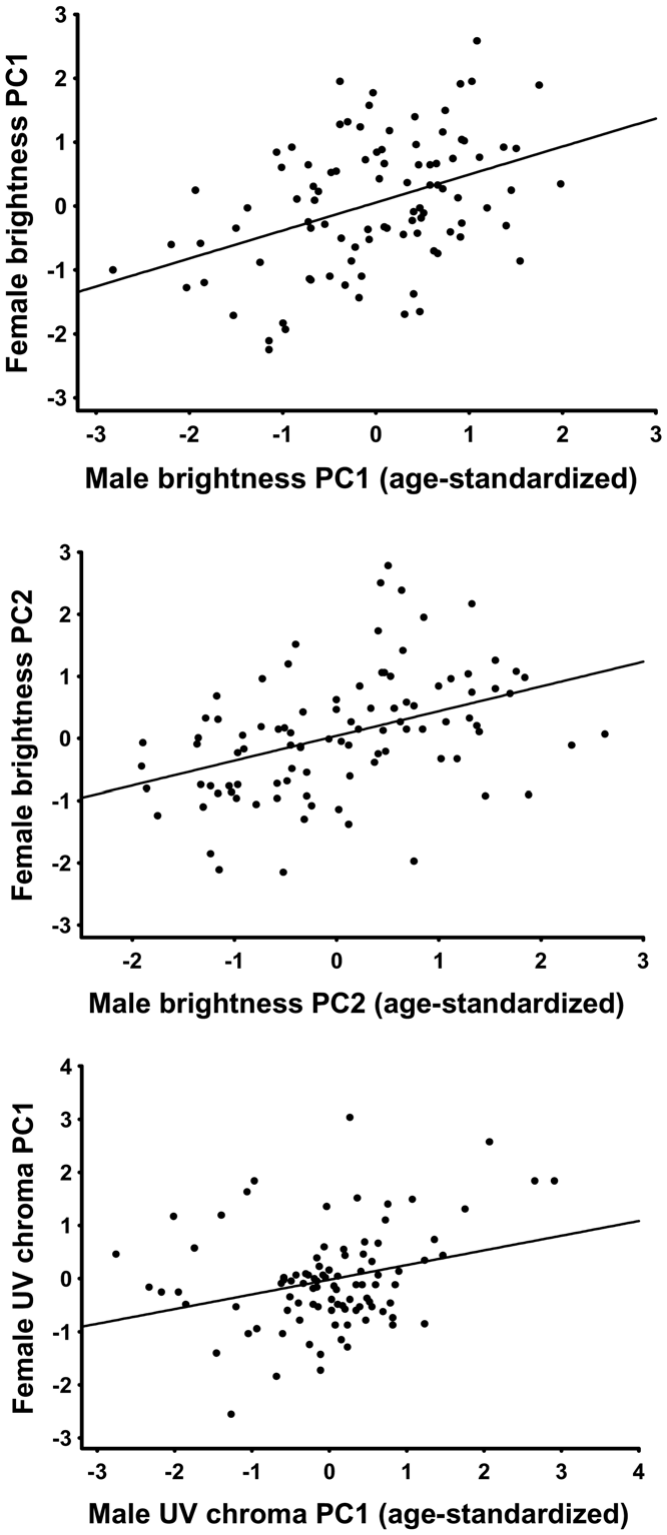
Assortative mating in breeding pairs with respect to composite measures of plumage coloration. Brightness PC1 and PC2 probably represent brightness variation resulting from feather structure and melanin content, respectively. UV chroma PC1 integrates the relative UV reflectance of the whole plumage.

The first UV chroma PC (UV chroma PC1) loaded positively with the UV chroma of each plumage area (Table 2). UV chroma PC1 therefore captures variation in the structurally based chromatic component of coloration of the whole plumage, independently of color type, i.e. a bird with higher UV chroma PC1 score has a plumage with more pronounced relative UV intensity (Fig. 3G–L, S–W). The eigenvalues of other PCs were smaller than one (results not shown). UV chroma PC1 did not correlate with brightness PC1 or brightness PC2 (p>0.5).

### Integration of ornamental patch sizes and spectral attributes

None of the color PCs showed consistent integration with wing or forehead patch sizes across the dataset (n = 252 for wing patch size and n = 127 for forehead patch size, see detailed results in Tables 3 and 4), but for brightness PC2 there was a significant three-way interaction between age, sex and wing patch size. The interaction of age×wing patch size was non-significant in females (F_1,119_ = 1.86, p = 0.18), but significant in males (F_1,122_ = 8.15, p = 0.005). Brightness PC2 correlated negatively to wing patch size in juvenile males (F_1,25_ = 9.25, p = 0.005), but it was uncorrelated in adult males (F_1,97_ = 0.47, p = 0.48). These models also confirmed the sex-dependent age effect on all three PCs. Adult males had higher scores for brightness PC1 and UV chroma PC1 but lower scores for brightness PC2 than juveniles, while color was not significantly age-dependent in females (details not shown). Relationships of color PCs with tarsus length were overall non-significant and often age-dependent, but the age-specific relationships were generally weak (details not shown).

**Table 3:** Relationships of spectral attributes with wing patch size in the collared flycatcher.

	Brightness PC1					Brightness PC2					UV chroma PC1				
	df	F	R	CIL	CIU	df	F	R	CIL	CIU	df	F	R	CIL	CIU
**Sex**	1, 237	4.25*	0.13	0.01	0.25	1, 238	2.24	0.10	−0.03	0.22	1, 243	1.54	0.08	−0.04	0.20
**Age**	1, 237	4.46*	0.14	0.01	0.26	1, 238	7.69**	0.18	0.05	0.29	1, 243	3.46	0.12	−0.01	0.24
**Body mass**	1, 237	0.001	0.00	−0.12	0.13	1, 237	0.27	0.03	−0.09	0.16	1, 242	1.06	0.07	−0.06	0.19
**Tarsus length**	1, 237	0.68	0.05	−0.07	0.18	1, 238	0.004	0.00	−0.12	0.13	1, 242	2.74	0.11	−0.02	0.23
**Patch size**	1, 236	0.08	0.02	−0.11	0.14	1, 238	7.84**	0.18	0.06	0.30	1, 242	0.47	0.04	−0.08	0.17
**Sex×age**	1, 237	11.56***	0.22	0.09	0.33	1, 238	12.24***	0.22	0.10	0.34	1, 243	6.61*	0.16	0.04	0.28
**Sex×body mass**	1, 237	0.77	0.06	−0.07	0.18	1, 236	1.15	0.07	−0.05	0.19	1, 241	1.18	0.07	−0.05	0.19
**Sex×tarsus length**	1, 236	0.84	0.06	−0.06	0.18	1, 237	3.26	0.12	−0.01	0.24	1, 241	0.17	0.03	−0.10	0.15
**Sex×patch size**	1, 235	1.67	0.08	−0.04	0.21	1, 237	1.56	0.08	−0.04	0.20	1, 241	0.28	0.03	−0.09	0.16
**Age×body mass**	1, 237	0.13	0.02	−0.1	0.15	1, 236	0.27	0.03	−0.09	0.16	1, 241	0.50	0.05	−0.08	0.17
**Age×tarsus length**	1, 237	4.07*	0.13	0.01	0.25	1, 238	8.15**	0.18	0.06	0.30	1, 241	0.01	0.01	−0.12	0.13
**Age×patch size**	1, 235	0.38	0.04	−0.08	0.16	1, 237	1.18	0.07	−0.05	0.19	1, 241	0.44	0.04	−0.08	0.17
**Sex×age×body mass**	1, 237	0.05	0.01	−0.11	0.14	1, 236	0.54	0.05	−0.08	0.17	1, 241	0.02	0.01	−0.11	0.13
**Sex×age×tarsus length**	1, 236	0.11	0.02	−0.10	0.14	1, 237	0.01	0.01	−0.12	0.13	1, 241	0.02	0.01	−0.11	0.13
**Sex×age×patch size**	1, 235	1.02	0.07	−0.06	0.19	1, 238	6.90**	0.17	0.05	0.29	1, 241	0.49	0.05	−0.08	0.17

Significant relationships are marked with bold (* p<0.05; ** p<0.01; *** p<0.001). CIL: lower limit of 95% confidence interval; CIU: upper limit of 95% confidence interval.

**Table 4:** Relationships of spectral attributes with forehead patch size in the collared flycatcher.

	Brightness PC1					Brightness PC2					UV chroma PC1				
	df	F	R	CIL	CIU	df	F	R	CIL	CIU	df	F	R	CIL	CIU
**Age**	1, 125	22.96***	0.39	0.24	0.53	1, 123	6.21*	0.22	0.05	0.38	1, 125	8.00**	0.25	0.07	0.40
**Body mass**	1, 124	0.32	0.05	−0.12	0.22	1, 122	1.65	0.12	−0.06	0.28	1, 124	0.10	0.03	−0.15	0.20
**Tarsus length**	1, 124	0.58	0.07	−0.11	0.24	1, 123	2.11	0.13	−0.05	0.30	1, 124	0.92	0.09	−0.09	0.26
**Patch size**	1, 124	0.31	0.05	−0.13	0.22	1, 122	3.12	0.16	−0.02	0.32	1, 124	0.53	0.07	−0.11	0.24
**Age×body mass**	1, 123	0.74	0.08	−0.10	0.25	1, 121	1.87	0.12	−0.05	0.29	1, 123	0.03	0.02	−0.16	0.19
**Age×tarsus length**	1, 123	0.85	0.08	−0.09	0.25	1, 123	6.93**	0.23	0.06	0.39	1, 123	0.01	0.01	−0.17	0.18
**Age×patch size**	1, 123	0.001	0.00	−0.17	0.18	1, 121	0.59	0.07	−0.11	0.24	1, 123	2.50	0.14	−0.03	0.31

Significant relationships are marked with bold (* p<0.05; ** p<0.01; *** p<0.001). CIL: lower limit of 95% confidence interval; CIU: upper limit of 95% confidence interval.

### Mating patterns for integrated color measures

For an ornamental trait that conveys similar information in the two sexes and functions in sexual selection in both sexes, we may expect positive assortative mating between the sexes [52]. This pattern was very robust in our breeding collared flycatcher pairs in relation to their plumage-level reflectance features. There was a positive correlation between the sexes for brightness PC1, brightness PC2 and UV chroma PC1 (r = 0.41, p<0.001, n = 95; r = 0.42, p<0.001, n = 95; r = 0.29, p = 0.005, n = 95, respectively; Fig. 3). In addition, there was a weaker positive relationship between female brightness PC1 and male UV chroma PC1 (r = 0.21, p = 0.040, n = 95). Other correlations were non-significant (all p>0.75). When looking at the confounders of assortative mating patterns, relative measurement date was unrelated to spectral reflectance (all p>0.06). Median laying date was negatively related to male UV chroma PC1 (r_s_ = −0.19, p = 0.037, n = 126), and positively to female UV chroma PC1 (r_s_ = 0.21, p = 0.016, n = 125), but unrelated to other PCs. Moreover, we detected very similar mating patterns when using residuals from regressions of the PCs on absolute measurement date (results not shown).

### Outline and predictions of the functional integration analysis

The detected, significant but moderate levels of color integration across the plumage are consistent with both the integrated and the separate use of area-specific color by the receivers, so further analysis was necessary to discriminate between these possibilities. If color cues from several different areas are processed together, we predict that assortative mating patterns will link these areas more strongly with each other than with other areas not involved in this common processing (see Introduction). We therefore assigned area-level assortative mating correlations to five groups based on their links to the two main color PCs: 1) within brightness PC1, the same individual spectral variable (i.e. variable A in males and variable A in females etc.), 2) within brightness PC1, different individual spectral variables (i.e. variable A in males and variable B in females etc.), 3) within UV PC1, the same individual spectral variable, 4) within UV PC1, different individual spectral variables, 5) between the two main axes (i.e. one individual spectral variable from brightness PC1 in one sex versus another variable from UV PC1 in the other sex). We then compared mean correlation strength between these groups. In case of common processing, we predicted no difference between 1) and 2) and between 3) and 4), but a significantly lower mean for 5) than for the other four groups. In case of separate processing, we predicted higher mean correlations in groups 1) and 3) than in 2), 4) and 5), with no difference among the latter.

In addition to the above comparisons, we assessed the normality of the frequency distributions of assortative mating correlations within brightness PC1, within UV PC1, and among the two PCs using Lilliefors tests to see whether these three can be considered as homogeneous groups without outlying individual correlations or groups of correlations. Homogeneity would be a sign of common processing of color variables within a given PC. Finally, we also conducted a sensitivity analysis and repeated the above tests by including under the relevant main axis a color variable that itself formed a single axis in that PCA. We expected this variable to disrupt both the relative magnitudes of mean effect sizes and the distribution of effect sizes for that main axis.

### Results of the functional integration analysis

**Figure 4:**
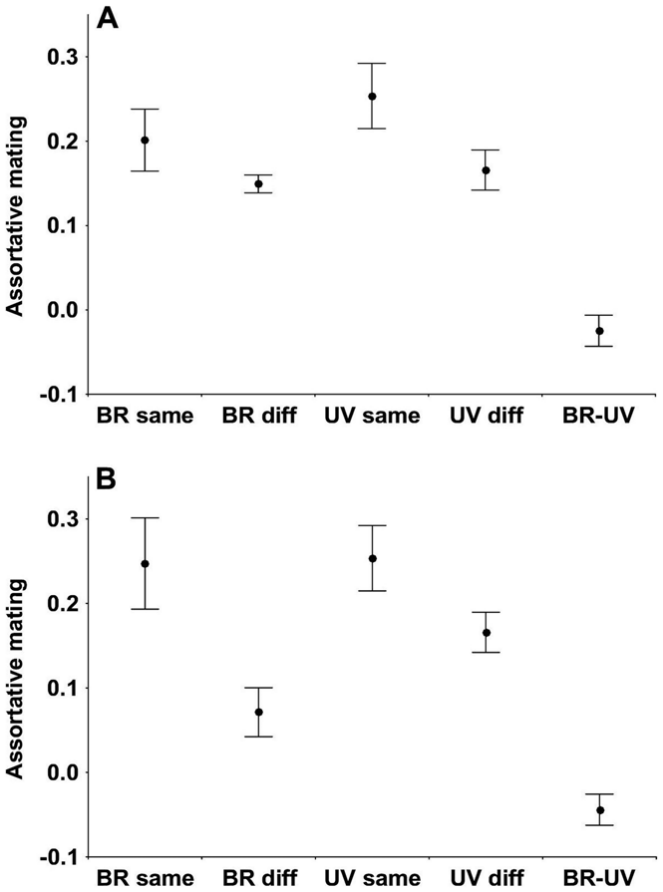
Assortative mating correlations in five different categories of trait pairs in collared flycatchers. Means±SE. In plot (A), the categories correspond to the integrated principal components (brightness PC1 and UV chroma PC1). In plot (B), the integration of brightness traits is disrupted by including the relatively independently varying wing covert brightness under brightness PC1. BR same, same brightness trait in males and females; BR diff, one brightness trait in males and another in females; UV same, same UV chroma trait in males and females; UV diff, one UV chroma trait in males and another in females; BR-UV, a brightness trait in one sex and an UV chroma trait in the other sex.

When omitting wing covert brightness (the only variable that loaded weakly in the first brightness PC) and using the remaining four brightness and five UV chroma traits, we found a significant overall difference among the the five groups of correlations (Fig. 4A; F_4,76_ = 20.10, p<0.001). This difference was solely because the among-axis group (one brightness versus one UV chroma trait) contained weaker correlations than the four within-axis groups (LSD tests, all p<0.0001), while the latter did not differ significantly from each other (p>0.061). The distributions of brightness-brightness, UV chroma-UV chroma and brightness-UV chroma assortative mating correlations did not deviate from normality (Kolmogorov-Smirnov d<0.138, Lilliefors p>0.05). These results are all consistent with the possibility that receivers integrate information from across the plumage in their mating decisions.

We tested the sensitivity of the functional integration test by also including the mating patterns of wing covert brightness, a trait that formed a separate brightness PC (PC2), as if it was part of brightness PC1. Based on the trait correlation matrix, this trait was not integrated with other brightness traits, so we also did not expect it to take part in functional integration. The inclusion of the assortative mating correlations of wing covert brightness biased the within-brightness, same-trait correlation group slightly upwards and the within-brightness, different-trait group strongly downwards, creating a similarly strong main effect as above (F_4,95_ = 18.33, p<0.001), but with significant pairwise differences between the within-brightness, different-trait group and the other three within-trait groups (all p<0.017). This indicates that individual brightness traits now played a significant additional role in mating over the integrated effect of the brightness trait complex. The among-axis group continued to deviate downwards from all other groups (all p<0.001; Fig. 4B), while all remaining comparisons were non-significant (p>0.160). The inclusion of the deviating brightness trait also disrupted the normal distribution of the brightness-brightness correlation group (d = 0.190, p<0.05), while the UV chroma-UV chroma and the brightness-UV chroma groups remained normally distributed (d<0.102, p>0.05). These findings indicate that the above results are not explained by the low power of the effect size comparisons, so the integrated treatment of color information by receivers may be a real phenomenon in this population.

## Discussion

In this study, we quantified the integration of spectral variation across five body regions in male and female collared flycatchers, and also examined the potential role of this variation in assortative mating patterns. Surprisingly, despite the readily apparent sexual dichromatism also visible in spectral attributes, we found that the main directions of individual color differences were statistically identical between the sexes. Moreover, we found robust positive covariation in reflectance descriptors across the plumage: the magnitude of covariation was similar in males, females and the pooled sample. Irrespective of their visible color, all measured areas showed significant parallel variation in UV chroma, and there were two dominant directions of variation in brightness. Finally, we found significant assortative mating for all three main color axes we detected, and mating patterns for individual spectral traits also suggested that trait complexes may exist which are treated together by the receivers. This grouping of the mating correlations is consistent with the idea that the plumage-level integration of spectral information has functional significance in this species. Our results have important implications for future studies of sexual selection on plumage color.

In species with multiple distinct color patches, ornaments have often been analyzed as separate characters without quantifying the interrelations of their color descriptors [57]–[61]. Other studies averaged measures of different plumage regions of the same color type to estimate overall plumage coloration [19], [62]–[65]. Averaging supposes absolute integration and ignores variation in relative color expression among areas. Few studies have used correlation matrices to justify treating different color cues as distinct ornaments [66] or as a single composite ornament [18], [51]. Here we used matrix comparisons and PCAs to devise composite spectral measures that quantify both positive and negative covariation between color variables (see also [18], [44]). Brightness and UV chroma were weakly interrelated, as also found in other species (e.g. [57], [67]). This independence could be due to their different proximate mechanisms, with brightness determined mainly by the amount of the light scattering and/or absorbing matter, while UV chroma influenced primarily by the regularity of feather microstructure [68]). We therefore treated these two spectral attributes separately.

Our matrix comparisons revealed that the correlation structures of brightness and UV chroma among plumage areas were shared between the sexes. The similarity of the patterns is interesting because there is strong sexual dichromatism, with female coloration being less pronounced, which may stem from the different selection pressures acting in the two sexes in terms of e.g. predation [69], [70] or sexual selection [71], [72]. In the case of overall sexual dichromatism, we might expect sex differences in the degree of integration among the component color traits as well [73]. However, it is possible that the proximate determination of plumage color expression is similar in the two sexes [10], which may have generated the detected similarity of sex-specific correlations in the collared flycatcher.

Coloration in collared flycatchers has two different origins (melanin- and structurally based “dark”, and purely structurally based “white” areas, see below). Intriguingly, brightness PC1 did not reflect variation in achromatic contrast between melanized and depigmented regions but instead it correlated in the same direction with all measured plumage parts (dark and white) except the wing coverts. As demonstrated in other bird species, structural light-scattering and pigmentary absorption may contribute together to light reflectance in the pigmented areas [6], [7], while only structural mechanisms can cause reflectance variation in depigmented white areas [5]. Based on this, we speculate that brightness PC1 may summarize variation in the structural component of intensity generated by light scattering, with individuals of high brightness PC1 scores having more reflective air-keratin tissue in the respective plumage parts. Such a structure also leads to more light transmitted through a similar amount of light-absorbing melanin, so that the brightness of depigmented and pigmented parts can change in the same direction. Unlike other melanized regions, the wing coverts appear to show high variation in visible brightness (light brown to blackish) in both sexes in our population, which also leads to large coefficients of variation for measurable brightness (our unpublished data). The added variation may stem from differential melanin deposition, which in turn may obscure the variation in brightness caused by feather structure. This could be one reason why brightness PC1 did not load with the wing coverts. Brightness PC2 correlated with only the two pigmented areas, but most strongly with the wing coverts, so it may represent the amount of melanin deposited. UV chroma PC1 indicated stronger integration across the plumage in spectral shape than in spectral elevation. The participation of wing coverts in the first PC in this case may be due to the strong effects of feather structure on UV chroma [7] overriding the weaker effects of melanins [74], [75]. The above speculations must be confirmed by studies of feather nanostructure in the collared flycatcher [5], but the interpretable color covariation patterns we detected illustrate the fruitfulness of examining spectral integration even among visually very distinct plumage areas.

We found little correlation between the overall color measures of the plumage and the sizes of ornamental white patches, which suggests that three different attributes of the plumage, brightness, UV chroma and patch sizes, may indicate different aspects of quality [3], [76]. As an exception, we did observe that brightness PC2 correlated negatively with wing patch size in young males. Feather wear on white patches may differ among individuals, particularly between sexes [31]. In our collared flycatchers, abrasion on the wing patch increases with wing patch size in young males, but not in older individuals, which suggests that the mechanical structure of these feathers may be weaker in young than in older males (G. Hegyi *et al.*, unpublished data). Smaller brightness PC2 in larger-patched young males suggests increased melanin content in wing feathers, which could be a compensatory mechanism to better protect at least the melanized parts of the wing against abrasion and breakage through the reinforcing effect of melanin deposition [24].

In our study, we found positive assortative mating with respect to plumage-level, composite axes of color. In other species, several studies have investigated mating patterns in relation to one ornament [77]–[79], while a smaller number of studies examined simultaneously several individual characters [14], [43], [80], [81]. To the best of our knowledge, no study has assessed assortative mating patterns with respect to overall, plumage-level color variation. Our findings do not seem to be due to the confounding variables age, body size or body mass, as age was corrected for in the analysis, body size was not involved in mating patterns (results not shown), while body mass was unrelated to our color PCs. Moreover, absolute or relative measurement date also did not seem to influence the mating patterns we found.

Mating patterns in relation to individual spectral traits may provide clues to tentatively answer the question of whether assortative mating for plumage-level color axes is due to receiver attention paid to each individual trait in the same direction, or due to the consideration of whole plumage reflectance as a single, integrated signal. First of all, we found little assortative mating between traits that did not contribute to the same color PC. This suggests that the trait complexes defined by PCs do have some role in mate acquisition. We also found that assortative mating within a single color trait (i.e. trait A in males versus females) was no stronger on average than assortative mating between two traits belonging to the same main color PC (trait A in males versus trait B in females). In addition, the normal distribution of effect sizes within a major category (within brightness main axis, within UV chroma main axis, between main axes) indicates the absence of traits or trait groups that deviate from this overall pattern. This strongly suggests that, if additional attention is paid to single-trait color attributes over plumage-level color attributes, this additional attention must be weak. In other words, the patterns we found are consistent with the coincindence of developmental and functional integration in plumage reflectance [82], [83].

Finally, we also examined whether this apparent coincidence was due to power issues that prevented the detection of small but important functional differences among traits within a single color axis. For this, we used wing covert brightness that seemed to form a largely independent second brightness axis. When nevertheless treating this trait as part of the developmentally integrated brightness trait complex, the resulting mating patterns showed evidence for additional individual trait effects for brightness but not for UV chroma. Moreover, the distribution of effect sizes for brightness was no longer normal, suggesting the presence of at least one trait that did not conform to the overall pattern. This indicates that our approach to mating correlations was capable of detecting deviations from functional integration, so our conclusions are robust.

Although our assortative mating results are not confounded by obvious background variables (see above), identifying the sexual selection pathway that generated these patterns will require experimental studies. Assortative mating could arise from mutual mate choice, and assortative mating and mutual mate choice were indeed simultaneously demonstrated in blue tits [78], [84] and rock sparrows [79], [85], [86]. An alternative mechanism is sexual competition in both sexes, potentially combined with female mate choice [87]. In our population, the sizes of white ornaments are known to play a role in intrasexual competition among both males [88] and females [89], which apparently creates ornament-related spatial settlement patterns in both sexes [72], [90]. It is therefore reasonable to assume that sexual competition in relation to plumage color contributed to the mating patterns we detected. Female preference direction may also depend on female phenotype [91], or choosiness may change with body condition, which in turn may correlate with ornamentation [92].

Finally, it must be noted that assortative mating assumes mutual sexual selection and it does not detect sexual selection acting on one sex only [52]. In the absence of detailed data on mating latencies after arrival from migration or the sampling strategies of individual birds, we were obliged to resort to this measure of sexual selection. However, using assortative mating correlations simplified our analyses in comparison to other measures of sexual selection. The ultimate clarification of sexual selection on these ornaments would come from separate manipulations of area-specific color parameters in different directions in a factorial design and mate choice experiments on the manipulated birds, but this would be a very demanding study in most wild birds, including our study species. In any case, these findings raise the possibility of sexual selection on correlated, composite ornamentation, which is highly relevant to the future of multiple ornamentation studies.

Researchers have increasingly realized the importance of multiple potential cues in the ornamentation of the same species [3]. This led to an atomistic approach that looks for sources of different information even within the same distinct sexual trait (e.g. [76], [93]). However, even the long-standing views regarding the distinct information content of different plumage color types are now being questioned [94], [95]. Moreover, it seems that different color-producing mechanisms act together and not in isolation in most cases [5], [6]. A recent study of great tits detected strong parallel variation, and assortative mating with respect to parallel variation, in two plumage areas of different color production mechanisms [44]. Our present results suggest that the spectral reflectance of the whole collared flycatcher plumage forms a moderately integrated system with similar axes of variation in males and females, despite the pronounced sexual dichromatism. Moreover, detailed mating patterns do not refute the idea that plumage-level spectral information may have a signal function in this species. If the color of different plumage regions of the same species is generally correlated and also used together [42], [45], then studies should increasingly focus on the joint variation of color in multiple plumage areas and its potential function in sexual selection. This paradigm shift will have important implications for the design and interpretation of both correlative and experimental studies [83], [96]. In collared flycatchers, future studies should examine the information content of overall plumage reflectance variation (i.e. condition-dependence and heritability [97]), and the mechanism underlying the assortative mating pattern (i.e. mate choice or sexual competition [87]).

## Materials and Methods

### Ethics statement

All work was conducted with a ringing license from the Hungarian Ornithological and Nature Conservation Society (MME, registration number 128), long-term research agreements with the Pilis Park Forestry (December 1988 and March 2007) and research permits from the regional nature conservation authority (KTVF 22021/2006, KTVF 43355-1/2008).

### Study site and species

This study was conducted in the breeding seasons of 2006, 2008 and 2009 in the Pilis Mountains, Duna-Ipoly National Park, Hungary (47°43′N, 19°01′E). The study site is a continuous deciduous woodland dominated by oaks, and consists of several nest box plots including ca. 800 artificial nest boxes used principally by collared flycatchers and great tits. The collared flycatcher is an insectivorous, sexually dichromatic, hole-nesting, long-distance migratory, single-brooded passerine [98]. The mating system is social monogamy with occasional polygyny and frequent extra-pair fertilizations [99]–[101]. The information content and function of the size of ornamental white patches are well-known in our population. Male forehead patch size is relatively less condition-dependent than male and female wing patch size [71], [72]. Male and female wing patch sizes, but not male forehead patch size, seem important in intrasexual competition [88], [89], but all three patch sizes seem to play a role in social mate acquisition [72], [102]. Plumage-level spectral features, on the other hand, have yet to be examined in this species. Coloration of the sister species pied flycatcher (*Ficedula hypoleuca*) shows a higher degree of individual variation in the human-visible part of the spectrum, and the UV component of plumage reflectance seems to be under sexual selection [63], [103], [104].

### Field methods

Birds were captured in the nest boxes when their offspring were 8 to 12 days old. Males were classified as yearlings or adults (>1 year) by their wing patch size and the darkness of primaries [53]. Female binary age was determined based on ringing data with unknown first breeders classified as yearlings [72]. We recorded the maximum width and height of the forehead patch (to the nearest 0.1 mm) using a calliper, and calculated forehead patch size by multiplying these two variables. Wing patch size was estimated as the sum of the visible lengths of white areas on the outer vanes of the fourth to eighth primaries on the right wing, also measured by calliper (to the nearest 0.1 mm). Tarsus length was measured by calliper (to the nearest 0.1 mm), and birds were also weighed by a Pesola spring balance (to the nearest 0.1 g). We visited the nest boxes three times a week to determine laying date, defined as the date of laying the first egg. In the analyses, we used the deviations from the median laying date of the respective year.

### Reflectance measurements

We measured reflectance of the plumage using an USB2000 spectrophotometer (range 179–877 nm; Ocean Optics Europe) with a Mini-DT2 deuterium-halogen light source (Ocean Optics Europe) in 2006 and 2008, and with a DH-2000 deuterium-halogen light source (Ocean Optics Europe) in 2009. We took reflectance spectra from the crown, forehead, wing patch, wing coverts and breast of the birds in both sexes. The bifurcated micron fibre-optic probe (R400-7; Ocean Optics Europe) comprised of six 400 µm illuminating fibres surrounding a 400 µm measuring fibre. The probe was oriented at a 90-degree angle to the plumage surface, and its tip fixed in a black plastic sheath to disbar ambient light and to standardize measuring distance (3 mm). The diameter of the measured area was 6 mm. Reflectance data were computed relative to a black standard and a white WS-1 diffuse reflectance standard (>98% refflectance from 200 nm to 2.5 µm) by the following formula: R = [(R_sample_−R_black standard_)/(R_white standard_−R_black standard_)]×100. The black (or dark) reference was measured while excluding all ambient light (i.e. no incoming light to the detector). The software (OOIBase32, Ocean Optics Europe) recorded the spectra in 0.37 nm steps and also calculated the relative reflectance data. We recorded two consecutive spectral readings for each plumage region in every bird, and re-measured the standards at regular time intervals to calibrate the system.

In the following analyses, we process the obtained spectral data using objective color descriptors and spectrum-level statistical analyses [105]. These analyses do not take the visual system of our study species into account. However, visual perception models (e.g. tetrahedral color space models [106] require, among others, information on ambient light color. Courting male collared flycatchers use at least two light environments with radically different ambient light spectra (woodland shade and small gaps [107]), and individual males seem to systematically choose a given light environment for courtship [108]. Therefore, visual perception modeling could be highly misleading in our case without knowing the light environment used by a given individual. Studies in this direction are currently underway in our population. Results for the objective color descriptors we present here are less specific to the study species than those from visual perception models, but they minimize the probability of major errors caused by unfounded assumptions (also see [105]).

**Figure 5:**
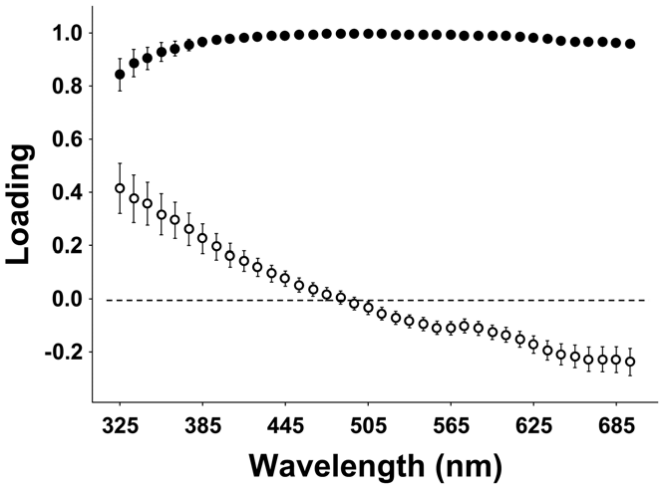
Average loadings of principal components of five plumage areas with wavelength bands as input variables. Means±SE. Filled circles, first PC; open circles, second PC. The principal component analyses were performed separately for each plumage area, and the sexes were pooled. Data were standardized for year and sex (to a mean of zero and a standard deviation of one) before the analyses.

From the raw reflectance spectra (i.e. without any averaging), we generated two objective color parameters for each body part. We calculated average intensity (brightness) from 320 to 700 nm (R_320–700_ [33], [109]), because this is the range of the light spectrum sensed by the majority of passerines [110] and because UV-manipulation experiments supported UV-sensitivity in the sister species pied flycatcher [103]. Brightness is an appropriate descriptor of achromatic intensity, regardless of the color type. The second color descriptor was relative UV reflectance (UV chroma), a standard gauge of plumage reflectance spectra, which describes the ratio of reflected UV light to total brightness (R_320–400_/R_320–700_ [33]). Principal component analyses of raw spectral information (following Cuthill *et al.*, 1999, details not shown here) yielded brightness and UV chroma as the two overwhelmingly dominant axes of spectral variation irrespective of the visible color of the respective area (Fig. 5), which supports our present treatment of the data.

To test the reliability of our spectral data, we estimated repeatability using Pearson correlations. Repeatability was calculated separately for each plumage area, both in females and males. It was high both for brightness (females: *r* = 0.82–0.97, all p<0.001; males: *r* = 0.76–0.99, all p<0.001), and for UV chroma (females: *r* = 0.82–0.92, all p<0.001; males: *r* = 0.61–0.97, all p<0.001), so we used the average of the measurements for each individual.

When we measured an individual in more than one year, and in the case of close relatives (parent and offspring, siblings), we randomly chose one data set and one individual, respectively, for the analyses. Moreover, we analyzed only individuals for which we had at least one set of complete reflectance data from each measured plumage region (n_yearling male_ = 28; n_adult male_ = 99; n_yearling female_ = 50; n_adult female_ = 75).

### Statistical methods

Spectral variables were standardized for year before any analysis (to a mean of zero and a standard deviation of one). Differences in plumage reflectance between females and males were analyzed by Student t-tests (Statistica 8.0, StatSoft, Inc.). We first used Pearson correlations to determine the relationships among the area-level color variables, separately in both sexes. To quantitatively assess the degree of color integration and the appropriateness of a plumage-level color analysis, we computed the KMO index of factor sampling adequacy [54], [111]. Higher values of the index correspond to higher ratios of shared variation, and PCAs are not recommended with KMO index values below 0.5 [54]. To compare the structure of the correlation matrices of brightness and UV chroma values between sexes, we used CPC analyses developed by Flury [56], as implemented in the program ‘CPC’ [55]. This method evaluates a hierarchy of models that represent different degrees of matrix similarity, from unrelated structure through different numbers of common PCs (CPC_1_ to CPC_k-2_, where k is the number of input variables) to situations where all PCs are similar and the relative or the absolute importance of the different PCs is also similar (matrix proportionality and matrix equality, respectively). The suitability of different similarity levels given the data is compared based on the balance of model fit and parsimony, using the Akaike Information Criterion (AIC). Models with smaller AIC values are considered better supported by the given data, but similar AIC values indicate that different similarity levels perform similarly.

As a second step, we used the CPC results to construct the PCAs from which we derived the plumage-level color descriptors used in the subsequent analyses. Following the Kaiser criterion, we included in these analyses only those principal components for which the eigenvalue was greater than one. We examined the relationships between PCs and ornamental patch sizes using general linear models, with backward stepwise model selection. One PC was used as a dependent variable in each model. When analyzing color in relation to wing patch size, we used age and sex as categorical predictors, and wing patch size, body mass and tarsus length as continuous predictors. We also tested the interactions of categorical predictors with continuous predictors, as well as the three-way interactions of sex, age and a continuous predictor. We used these three-way interactions because age-effects on color differed between the sexes (see “Results”). When the focal covariate was forehead patch size, we did not use sex as a categorical predictor because this patch is consistently present in males only. We included the two-way interactions of age with the continuous predictors.

Assortative mating pattern in relation to plumage coloration was examined by Pearson correlations. Before analyzing mating patterns in relation to composite measures of coloration, we standardized the PCs of males for age (to a mean of zero and a standard deviation of one) because male color was age-dependent (see “Results”). We then constructed an *n*×*n* correlation matrix of the main color axes of males versus females (where *n* is the number of main axes). The mating pattern may be influenced by the association of the PCs with laying date or relative measurement date (deviation from laying date), so we also assessed the relationships between the PCs and absolute or relative date using Spearman rank correlations. Direct correction of mating patterns for date using general linear models brought very similar results regarding mating patterns, but this approach is not reported here because of the non-normal distribution of the date variables.

To examine whether the detected assortative mating for an integrated, plumage-level color axis (i.e. one integrating the five measured plumage areas) was due to sexual selection on plumage-level or rather area-level color, we also examined assortative mating correlations in an *n*×*n* matrix of individual spectral variables of males versus females (where *n* is the total number of different brightness and UV chroma traits for individual plumage areas). Variables were standardized for year in females and for both year and binary age in males. We used the raw Pearson correlation values to test the predictions outlined in the Introduction in a meta-analytic approach (using Fisher's Z transformation of *r* yielded exactly the same results). We used a general linear model to compare five categories of effect sizes, and Lilliefors tests to assess the normality of the distribution of r in three larger categories. The details of these procedures and their sensitivity tests are described in the Results section.

Before any parametric test, we tested the frequency distributions of all variables for normality by Lilliefors tests. We used an alpha level of 0.05 and two-tailed significance tests throughout.
